# Painted playgrounds for preschoolers’ physical activity and fundamental motor skill improvement: a randomized controlled pilot trial of effectiveness

**DOI:** 10.1186/s12887-023-04260-2

**Published:** 2023-09-09

**Authors:** E. Kipling Webster, Maura M. Kepper, Sanjoy Saha, Robbie A. Beyl, Chelsea L. Kracht, Jessica St. Romain, Amanda E. Staiano

**Affiliations:** 1 Department of Kinesiology, Recreation, and Sport Studies, University of Tennessee, 1914 Andy Holt Ave, Knoxville, TN 37996 USA; 2 Washington University of St. Louis, One Brookings Drive, St. Louis, MO 63130 USA; 3 Pennington Biomedical Research Center, 6400 Perkins Road, Baton Rouge, LA 70808 USA; 4 Virginia Commonwealth University Health, Sanger Hall 1101 E. Marshall, Richmond, VA 23298 USA

**Keywords:** Children, Exercise, Preschool, Early childhood, Playground

## Abstract

**Background:**

Preschool children are not meeting recommended levels of physical activity (PA) nor are they proficient in fundamental motor skills (FMS), which are the foundation for PA. As such, interventions are needed to increase PA and FMS in young children. This trial examined the effects of an environmental (“painted playgrounds”) and capacity-building (written toolkit) intervention on child FMS, PA, and sedentary behavior at early childhood education (ECE) centers and examined feasibility.

**Methods:**

In a randomized controlled trial, four ECE centers were randomly assigned to an intervention group or wait-list control. For intervention centers, stencils were spray painted adjacent to playgrounds and teachers were provided material for using stencils for FMS practice. Follow-up assessments were conducted six to eight weeks after baseline. Time spent in PA and sedentary behavior was assessed via accelerometry and FMS were evaluated using the Test of Gross Motor Development (TGMD-3) at baseline and follow-up. A repeated measures linear model was performed to test the effects of the painted playgrounds on the primary outcomes of interest. Feasibility was measured by stencil engagement via direct observation and satisfaction surveys.

**Results:**

A total of 51 preschoolers completed baseline assessments (4.3±0.6 years; 43.1% male). There were no significant changes in PA or sedentary behavior (all confidence intervals contain 0) between control and intervention groups. Intervention children significantly improved ball skill, locomotor, and overall TGMD-3 percentile scores at follow-up (all (all confidence intervals contain 0), which was not observed in control group. However, there was no significant change in FMS between the control and intervention groups (confidence intervals contain 0). For stencil use, boys and girls interacted with different stencils during their free play. Directors and teachers reported children incorporated academic concepts and initiated games, and teachers prompted more PA opportunities on the playground.

**Conclusions:**

This intervention did not show statistically significant changes in children’s PA, FMS, or sedentary behavior compared to a control group; however, small FMS improvements for the intervention group were found from baseline to follow-up. Further work should examine intervention fidelity as well as inexpensive supplies, teacher training, or other strategies to increase preschool children’s PA and improve FMS at ECE centers.

## Background

Few children ages three to five years meet the World Health Organization (WHO) recommendations of at least 180 min/day of physical activity (PA), of which at least 60 min are moderate-to-vigorous physical activity (MVPA), and limit sedentary time, typically measured as one hour of sedentary screen-time [1, 2]. This low adherence has implications for higher excess weight gain, along with limitations to physical abilities, with life-long implications. A critical aspect of PA participation for children is the development of fundamental motor skills (FMS) that allow for engagement in a variety of PA tasks and are the foundation for more advanced movements [3], for confidence, [4] and for continued engagement in physical activity [5–7]. Failing to provide adequate opportunities for young children to engage in sufficient PA can deprive them of the basic skills needed to be physically active throughout their lives.

To improve preschooler PA and FMS and to reduce time spent sedentary, it is important to consider where preschoolers spend their waking hours. Maximizing time spent in MVPA during outdoor activities is a key strategy, particularly outdoor play at formalized early childhood education (ECE) centers which are attended by 80% of preschoolers in the United States [8]. Though the outdoor play setting is the primary place where most PA occurs at the ECE center, preschoolers are sedentary for over half of their time outdoors [9]. Consequently, past research promoting preschoolers’ PA and FMS have targeted these outdoor settings to increase the amount of time children spend physically active while outdoors [10, 11]. These programs have employed various approaches including a peer coach who helps ECE teachers embed physical activity throughout the daily curriculum [12], implementing shorter and more frequent outdoor play periods [13], and changing the outdoor play setting by adapting or adding moveable (portable) and unmovable (fixed) parts [14]. These programs have reported mixed results in improving preschoolers’ PA and FMS [13, 15]. A review paper summarized that only eight of 18 studies (conducted in ECE centers with PA as the main outcome) reported significant improvements in PA [16]. Chief barriers to implementation and effectiveness include limited teacher training to support sustained PA coupled with the high cost of upgrades to the playground/outdoor setting [17].

A combination of environmental and capacity-building interventions may overcome limitations in teacher capacity and translate to more PA over the long-term. Adding colorful markings (e.g., hopscotch, foursquare) to existing outdoor play settings and open spaces may provide a low-cost, feasible way to increase children’s PA outdoors while also providing teachers with structured opportunities to help children build motor skill proficiency. These markings can be easily applied and implemented in outdoor play settings, since they use existing open space (e.g., concrete or other hard surface), have a low-cost for application, and can be sustained when using long-lasting materials (e.g., spray paint). These “painted playgrounds” have resulted in an increase in MVPA in elementary age children in outdoor play sessions [18, 19], yet have not been tested in younger children attending ECE centers.

Stencils are a viable option to promote PA and FMS in this age range as they can facilitate higher intensity PA and locomotor skills (e.g., jump, hop, skip), through activities like hopscotch or jumping between places [20]. Therefore, the primary aim of this pilot study was to test the preliminary effectiveness of a playground stenciling intervention with brief teacher instruction to increase FMS, PA, and reduce time spent sedentary among preschoolers attending ECE centers. It was hypothesized that children attending ECE centers that received stenciling and educational materials would increase minutes of MVPA, decrease minutes of sedentary time, and improve FMS scores after the intervention and compared to those attending ECE centers assigned to a waitlist control. The secondary aim was to examine the feasibility including director satisfaction and the time and cost of stenciling.

## Methods

The “Painted Playgrounds” was a randomized controlled trial study occurring in four ECE centers (two intervention; two waitlist control). The centers were identified using a public list assembled by the state Department of Education comprised of licensed ECE centers within a metropolitan county of a southeastern U.S. state. ECE centers were eligible if they were currently licensed, enrolled at least 20 children between the ages of three to six years, and had available concrete area within their outdoor play environment to allow for stenciling three designs. Trained researchers reviewed the outdoor play setting and assessed eligibility of available area for stenciling (i.e., square footage and surface substrate). If ECE centers were eligible, written approval and consent were obtained from the ECE center’s director/administrator to allow for the playground stenciling and playground observations.

Preschoolers at these ECE centers were eligible if they were between the ages of three to six years, enrolled full-time (at least six hours/day, five days/week), and planned to attend the same ECE center for the study duration (the following eight to nine weeks). Parents/guardians of preschoolers provided written consent for the child measurements (i.e., height, weight, accelerometry) and were provided with an opt-out letter as passive consent for the playground observation. The study was also explained in a child-friendly manner to participants, and preschoolers were given the opportunity to refuse measurements. The study protocol was approved by the Institutional Review Board (IRB) of Pennington Biomedical Research Center.

## Procedures

ECE center directors were asked to complete surveys related to their ECE center characteristics. Informational materials were distributed to parents about the study and to obtain written consent. The full purpose of the study was not disclosed to the director, teachers, parents, or preschoolers at the ECE centers until after the completion of all measurements and observations to minimize the alteration of the teachers’ and preschoolers’ behaviors. Once parents provided consent, they were asked to complete and return a child demographic questionnaire. After consent and parent survey were obtained, the four centers were randomized to condition by a biostatistician. At the baseline visit, a trained researcher measured preschooler height, weight, and assessed FMS. Preschoolers were asked to wear accelerometers continuously for 24 h/day over seven days.

Time spent in the outdoor play setting was video recorded at all four ECE centers. Observations were rescheduled in inclement weather. One week after the baseline visit, playground stencils were painted at the two intervention ECE centers. The follow-up visit occurred approximately six to eight weeks after the baseline visit at all four centers. All measures were repeated including the seven day accelerometry protocol. Stenciling was conducted at the two wait-list control centers after the follow-up visit. Follow-up surveys were administered to ECE directors to examine satisfaction and perceived usefulness of the stencils. Process measures (e.g., time and cost of stenciling) were used to examine feasibility.

### Intervention

**Fig. 1:**
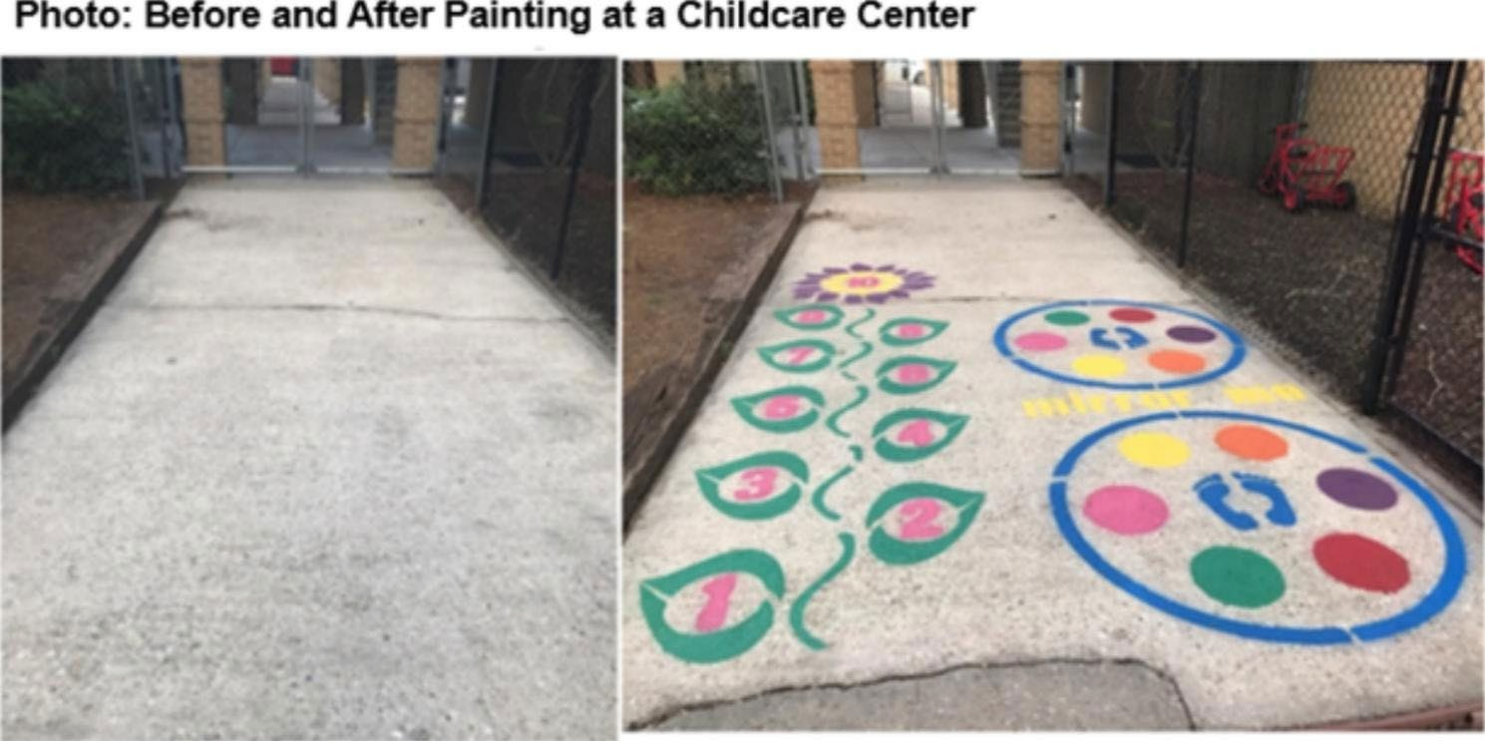
Hopscotch Sunflower (left) and Mirror Me (right) Stencil

**Fig. 2:**
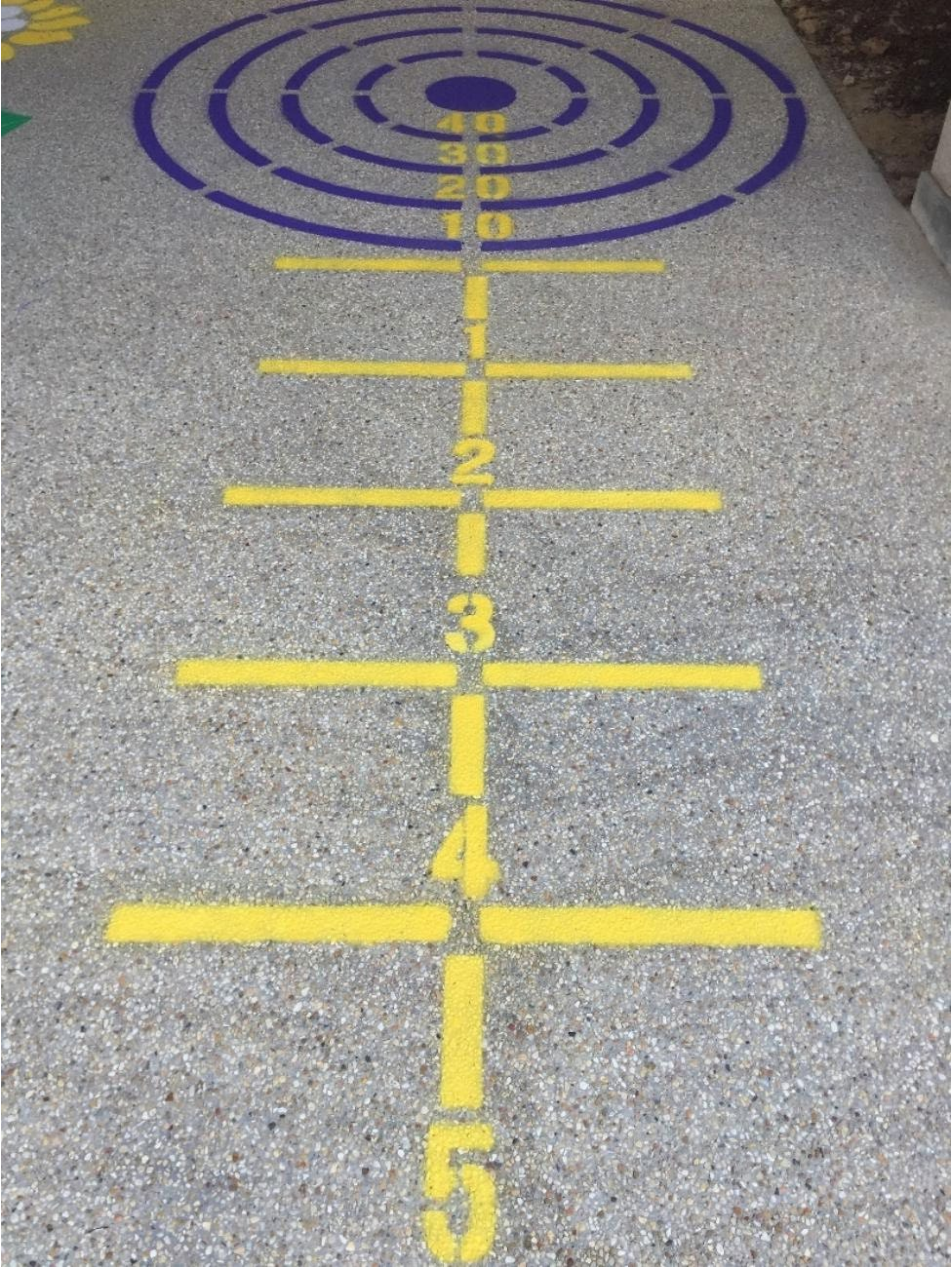
Bull’s Eye Stencil

Stencils were spray painted onto an open concrete area within the outdoor play setting for preschoolers (ages three to six years) at the ECE center. Stencils were purchased from Fast Line Striping Systems (Kingston, Ontario, Canada) including Sunflower Hopscotch (US $159), Bull’s Eye (US $179), and Mirror Me (US $149; See Figs. 1 and 2). Stencils were chosen by a kinesiologist and a developmental psychologist based on their potential ability to promote locomotor skills (e.g., jump, skip, and hop) and object control skills (e.g., underhand toss, overhand throw), the age and developmental stage of preschool-aged children, and feasibility of implementation (e.g., overall size of the stencil). The total cost of spray paint for the stencils (6 to 7 colors) per ECE center was approximately US $200. One day prior to stenciling, two staff members spent approximately two hours preparing the concrete or hard surface by sweeping and pressure washing. Stenciling took an additional two to three hours on a separate day, with two staff members applying two to three coats of spray paint. The stenciling process was tested prior to the intervention in a comparable environment to the ECE centers. Stencil placement and design were approved by ECE center directors, and stencils were painted in areas that would not interfere with existing equipment in the outdoor play setting or near falling hazards such as curbs, tree roots, benches, or doorways. These stencils were primarily painted in a fenced-in outdoor area outside of the main doorway, thus were subject to both shade and sun.

A toolkit (written booklet) was developed that provided childcare center staff with step-by-step instructions and photos to describe developmentally appropriate activities/games for their students during play using the stencils. With the toolkit, an expert (EKW) in motor skill development and PA in pediatric populations and two school physical education teachers developed a short video (11 min) for ECE center staff. This video included information on the importance of FMS as well as strategies/games and examples for using the playground stencils. The videos included preschool-aged children modeling how to use the stencils, to help teachers utilize the stencils during outdoor time to help children perform appropriate movements and facilitate the execution of proper FMS. The video and written toolkit complement each other to provide a complete package for ECE center staff to use stencils appropriately and easily during outdoor play to encourage safe and fun activities for preschool children with limited to no additional equipment required.

Thirteen printed toolkits were delivered to the two intervention ECE centers so that each teacher and director would have their own copy during the intervention period. The video was delivered to intervention ECE centers on a jump drive and was also sent to each director electronically via email with a link directly to the toolkit video on a private YouTube channel. The director was asked to watch the video and share it with all preschool teachers. A study staff member spent a total of approximately two hours at each intervention ECE center (spread across two to three visits) to meet with the director and lead teachers to explain the project, to present the video to the teachers, and to answer questions. The staff member also provided contact information to the teachers in case they had questions. After data collection ended, the study staff painted the same stencils on the playgrounds of the control ECE centers and also distributed toolkits and the video to the staff of the control centers.

### Measures

#### ECE center and preschooler characteristics surveys

Prior to baseline measurements, ECE directors completed a questionnaire related to policies and programs within their center. These questions were based on the Nutrition and Physical Activity in Child Care Survey, a widely used valid and reliable self-report questionnaire to assess nutrition and PA practices in ECE settings [21]. Questions included the amount of time allowed for free play and amount of time spent outside (including exact outdoor play times for the week of evaluation), structured programs and/or field trips that allow children to be physically active (including number of times per day or week), if one or more community organizations support or provide PA programs at the center, and whether the ECE center had policies regarding television viewing and computer use. Additionally, the study team directly observed and documented playground equipment (portable and fixed) for each center. The directors repeated the survey at the end of the study.

#### Anthropometry

Preschooler height and weight was assessed at both baseline and follow-up visits. Preschooler height and weight was measured in a private setting, dressed in light clothing and shoes removed. A trained researcher measured height and weight to the nearest 0.1 unit (cm and kg, respectively), and a third measure was taken if measurements differed by more than 0.5 units. Body mass index (BMI) z-score was calculated based on the age and sex specific national growth standards [22].

### Effectiveness

#### Physical activity and sedentary time

PA, time spent in MVPA, and sedentary behavior were measured by a triaxial accelerometer (Actigraph GT3X+, Ft. Walton Beach, FL). Children were measured 24 h/day for seven days. Measurements occurred for one week at baseline (before stenciling) and follow-up (six to eight weeks after stenciling). The child was outfitted with the accelerometer on an elasticized belt, on the right mid-axillary line by trained research staff and returned the accelerometers seven days later to the ECE program. During wear weeks, study staff checked accelerometry wear during school hours to assess for compliance. Based on previous work, [23] the minimal amount of accelerometer data that was considered acceptable was a two-day school wear time with a start and stop time of 8:30 am to 3 pm to account for average ECE scheduling. The research team verified the data for completeness using ActiLife software (version 5.6 or higher; ActiGraph, Pensacola, FL). Cut points were assigned based on Pate [24]. Minutes of light PA, moderate PA, and vigorous activity PA, MVPA (sum of moderate and vigorous PA), and sedentary time were calculated as an average across the wear days for children.

#### Fundamental motor skills

The Test of Gross Motor Development-3rd edition (TGMD-3) was used to assess preschooler ball and locomotor skills. The TGMD-3 is an assessment that uses systematic observation to evaluate children’s FMS using both the process- and product-oriented performance criteria, and has been validated within children ages three to 10.9 years [25]. The TGMD-3 assesses 13 FMS skills including locomotor (running, horizontal jumping, hopping, skipping, sliding, galloping), and balls skills (two-hand striking, one-hand striking, catching, kicking, overhand throwing, underhand throwing, and dribbling) [26]. Preschoolers were assessed at the ECE center using a group format, whereby groups of approximately three to four preschoolers completed the assessments together. A trained researcher would demonstrate the proper execution of the skill, and then each preschooler was allowed one practice trial then two formal trials which were used for evaluation. These trials were video recorded and then coded by trained researchers who had established > 95% reliability with an expert coder (intra-class correlation: 98%). A raw score was calculated using both graded trials and summed for both locomotor and ball skills. Raw scores were transformed into percentile scores according to manual guidelines [26].

### Feasibility

#### Stencil use and engagement

Engagement with the playground markings was evaluated through an adapted version of the System for Observing Play and Leisure Activity in Youth (SOPLAY) tool, which is a valid and reliable measure [27–29] created to systematically evaluate PA and energy expenditure in a targeted area through a momentary time sampling protocol [27]. For this protocol, the aim was to capture how frequently the stencils were being used on the playground. Like SOPLAY, the research staff established zones that encompassed the three playground markings on the playgrounds and every minute the three zones were systematically scanned to tally the number of boys and girls playing in each space and engaging with the marking along with field notes on the type of activities. This process was repeated for a 30-minute outdoor recess time, and totals were summed at the end of each period.

Engagement was defined as actively playing on the painted marking, waiting in line to interact with the marking, or being active in proximity with those on the marking (e.g., hopping alongside another child hopping on the marking). Trained research staff conducted this assessment prior to the follow-up measures being conducted to allow for reactivity to dissipate for the use of the playground stencils; live and video recorded the sessions for reliability checks. Each research staff attended the ECE center for one day and observed a 30-minute outdoor recess in the morning and a 30-minute session in the afternoon, totaling one hour of observation at each center. Prior to scoring, a research assistant was trained to implement the adapted SOPLAY and attended two trial observations prior to observing outdoor time for this pilot study and established > 90% reliability in the protocol and maintained this through video recorded reliability checks on 50% of the data.

#### Satisfaction with training and stencils

Directors of each intervention center completed a follow-up survey that included open-ended questions. The survey assessed satisfaction with and usefulness of training materials. Directors were asked to describe whether or not, and if so, how: (1) stencils were used to teach academic concepts, (2) teachers used stencils to increase PA, and (3) stencils changed students’ activity. Director surveys were also used to gather qualitative information for implementation feedback (e.g., what changes they would make to training materials, recommendations for future stenciling).

### Statistical analysis

The primary endpoints were change in school-day PA (light, moderate, and vigorous) and sedentary time measured by accelerometry. The secondary outcome was change in FMS percentile scores. Feasibility outcomes include stencil engagement observations to assess which stencils encouraged the most PA for boys and girls and director satisfaction and planned sustainment of the stencils. Statistical analyses were performed with the latest version of SAS, version 9.4. Due to the nature of this pilot study, the statistical focus will primarily be on confidence intervals. The level of significance for statistical analyses was 0.05 for demographic related variables and 95% confidence intervals not containing zero (CIs ∉ 0) for outcome related variables. The repeated measure linear model estimates the least square means for each time and group combination, no other covariates included. These least square means are used to determine whether changes in outcome variables were different between the intervention and control ECE centers across time. The models are used on dependent continuous variables including light PA, moderate PA, vigorous PA, MVPA, sedentary behavior, ball skill percentiles, locomotor skill percentiles, and total TGMD-3 percentiles. The linear model were used to determine whether changes in outcome variables were different between the intervention and control ECE centers. For all outcomes that used statistical models, all participants with at least baseline values were included in analyses; therefore, sample size varied based on the analysis. Directors’ satisfaction survey was presented in frequency and responses of open-ended questions were examined to identify common themes. For this pilot study, we set power to 80% and alpha = 0.05. With a sample size of 40 total participants, this study would need to observe effects sizes of at least 0.91 to obtain statistically significant results.

## Results

In total, 190 ECE centers were contacted, 101 ECE centers completed phone screening, and ultimately four ECE centers participated (two ECE centers/group). The ECE centers that did not enroll were unwilling to participate (n = 4), did not offer full-time enrollment (n = 2), did not have enough preschoolers (< 20) enrolled (n = 39), and 56 ECE centers did not have adequate concrete space to paint at least three stencils within their ECE center.

The four centers that enrolled included two Head Start centers, one church-affiliated center, and one hospital-affiliated center. One Head Start was randomly assigned to each condition. The centers averaged 184 children enrolled ages zero to five (range: 90–300) and 104 in the target age range of three to five year olds (range: 30–180). Directors reported 100% of children received state funding for meals in three of the four centers, with the fourth center reporting no state funding for meals. All four centers reported being in operation for 10 years or more and being active participants in the state star (i.e., quality) rating system for ECE centers. Two of the four centers also participated in additional accreditation, with one in each condition.

During the baseline interview, directors of all four centers reported providing at least two hours of free play each day and at least 60 min of active play time per day. Two directors reported allowing “no more than 15 minutes at a time” of continuous seated time each day and two allowed “15–30 minutes but only 1 occasion” per day. All four center directors reported providing “multiple play areas, open space for running, and a track/path for wheeled toys” with “good” or “lots of” variety both indoors and outdoors. Two of the four centers offered structured programs/field trips for PA and three offered community organizations who came to the center to provide PA. All four directors reported not withholding active play time for misbehavior. Directors reported providing one or two trainings per year for teachers and one or two trainings per year for parents on PA education, and all four directors reported having a written PA policy. There were no appreciable differences in the directors’ responses to questions at the end of the study period.

For the child-specific measurements that required parental consent, parents of 55 preschoolers returned consent forms, which included 33 preschoolers at intervention ECE centers and 22 preschoolers at wait-list control ECE centers. Of the 55 who completed consent forms, 54 completed baseline assessments, and 51 (n = 32 intervention, n = 19 control) completed follow-up assessments that occurred six to eight weeks after baseline assessments. The study took place between December 2018 – June 2019.

**Table 1:** Baseline demographics of participants by group

	Total (*n* = 51)	Control (*n* = 19)	Intervention (*n* = 32)	*P*-value
Age in years (SD)	4.3 (0.6)	4.6 (0.7)	4.2 (0.6)	0.03*
Male (%)	22 (43.1)	7 (36.8)	15 (46.8)	0.25
Race (%)				0.74
White	13 (25.5)	6 (31.6)	7 (21.9)	--
African American	35 (68.6)	12 (63.2)	23 (71.9)	--
Others	3 (5.9)	1 (5.3)	2 (6.3)	--
BMIz (SD)	0.5 (1.2)	0.4 (0.9)	0.6 (1.45)	0.52
Weight Status (%)				0.75
Normal	39 (76.5)	15 (79.0)	24 (75.0)	--
Overweight/Obesity	12 (23.5)	4 (21.1)	8 (25.0)	--

SD = standard deviation; BMIz = Body Mass Index z-score; *p < 0.05

As shown in Table 1, this sample was on average four years of age and predominantly African American (69%). About one-quarter of the preschoolers had overweight or obesity. Preschoolers in the control group were significantly older than preschoolers in the intervention group (*P* = 0.03). No other significant differences in demographic characteristics were observed between the groups (*P*s > 0.05).

### Physical activity and sedentary behavior

For PA and sedentary behavior measurements, wear time restrictions resulted in having different sample sizes at baseline and follow-up for school-day activity (Table 2). For school-day activity, there were no significant changes in PA within the intervention group during the observation period (CIs ∈ 0). Children in the control group significantly increased their vigorous PA (mean change score: 2.6 min/day, (0.1, 5.1) ) during the observation period. During school-time, children in the control group engaged in more minutes of light PA, moderate PA, vigorous PA, and MVPA at both baseline and follow-up compared to those in the intervention group; however, the differences of mean change scores between groups were not different (CIs ∈ 0). The mean change score of sedentary behavior (mean: -8.5 min/day, (-22.2, 5.2)) was high in control group compared to the mean change score in intervention group (mean: -5.6 min/day, (-17.9, 6.6)). The difference of mean change score of sedentary behavior was not significant between two groups (CIs ∈ 0) during the observation period.

**Table 2:** Changes in minutes of physical activity and sedentary behavior by group^

Weekdays during school-time	Control				Intervention			
	Baseline (n = 19)	Follow-up (n = 17)	*CI*		Baseline (n = 26)	Follow-up (n = 22)	*CI*	CI*
Light PA	48.3 ± 3.7	50.6 ± 2.9	(-4.3, 9)		41.8 ± 3.1	46.1 ± 2.5	(-1.6, 10.2)	(-3.2, 16.1)
Moderate PA	30.9 ± 3.8	34.4 ± 3.6	(-3.7, 10.7)		26.9 ± 3.2	27.3 ± 3.1	(-6, 6.8)	(-6, 14)
Vigorous PA	5.8 ± 0.8	8.4 ± 1.3	(0.1, 5.1)*		4.5 ± 0.7	5.4 ± 1.1	(-1.3, 3.1)	(-0.9, 3.5)
MVPA	36.7 ± 4.5	42.9 ± 4.4	(-2.1, 14.5)		31.4 ± 3.8	32.8 ± 3.8	(-6.1, 8.8)	(-6.5, 17.1)
Sedentary Behavior	305.0 ± 7.8	296.5 ± 6.7	(-22.2, 5.2)		316.8 ± 6.5	311.2 ± 5.8	(-17.9, 6.6)	(-32.2, 8.6)

*^Assessed using Actigraph GT3X + controlling for child’s age, sex, and accelerometer wear time;*

PA = Physical Activity; MVPA = Moderate-to-Vigorous Physical Activity; *CI for the changes between two groups

*Estimates are based on the results from the linear mixed effect model.*

### Fundamental motor skills

**Fig. 3:**
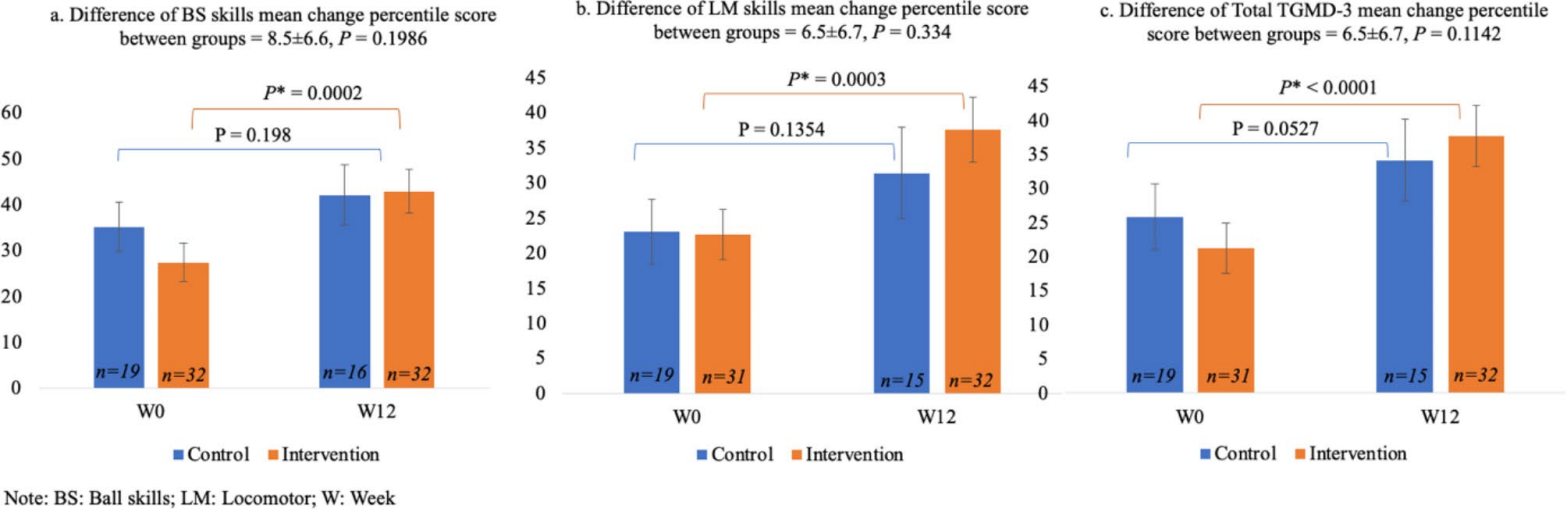
Comparison of **(a)** ball skills, **(b)** locomotor skills, and **(c)** total TGMD-3 percentile scores between control and intervention groups

Data were analyzed for the participants who completed either baseline or follow-up assessments, and thus, there were different sample numbers for ball skill percentile, locomotor skill percentile, and total TGMD-3 percentile scores (Fig. 3). There were no significant baseline differences between the two groups for the variables of interest (ball skill percentile, locomotor skill percentile, and total TGMD-3 percentile scores). Preschoolers from the intervention group had significant improvements in ball skills, locomotor skills, and total TGMD-3 percentile scores during follow-up compared to baseline (*CIs*∉ *0*); there were no changes in FMS in the control group (*CIs* ∈ 0). The means of total TGMD-3 percentile score at baseline in control and intervention groups were similar (25.8 vs. 21.2), and after the intervention, the TGMD-3 total percentile score means increased (34.1 and 37.7). However, when comparing intervention and control, there was no significant difference (CIs ∈ 0) in change in preschoolers’ FMS between groups (Fig. 3).

### Stencil use and engagement

Over two hours of observation across both intervention centers, in total 150 children played with the sunflower hopscotch, 49 children played with the “mirror me” stencil, and 37 preschoolers with the “bull’s eye” stencil during outdoor play. The sunflower hopscotch was more popular among boys; 73% (*n* = 109) of the children who played with the sunflower hopscotch were boys. The “mirror me” stencil equally attracted both boys (49%, *n* = 24) and girls (51%, *n* = 25). The bullseye stencil was more popular among girls; 68% (*n* = 25) of the children who played with the bullseye were girls. During the playground marking observations, it was noted that most of the activity was child-led, although teachers did prompt use on two occasions and children created their own games to engage with the markings and incorporated academic concepts (e.g., counting). The majority of the observations indicated that children were engaging with the markings in small groups of two to four children (70.4%), however group sizes ranged from two to seven; individual play was observed 15.4% of the time.

### Satisfaction with training materials and stencils

Overall, the toolkit, video and stencils were positively received by the directors and teachers at the intervention centers (see Table 3). Follow-up assessments with directors indicate that all three stencils generated interest and activity from children at both centers. One center stated that all teachers of three to five year-olds were using the stencils regularly and enjoyed having additional resources available to them on the playground. A director also mentioned that while the teachers were not always available to lead a small group in a stencil activity, the children still used them to play by making up their own games (sometimes with supplies like bean bags and other times with no supplies just their imagination). Children were seen using the stencils as markers to run, jump, bike, etc. One center director reported that the teachers use playground stencils to teach academic concepts, such as colors and numbers. The same center noted that they are interested in having more stencils painted to better use their concrete space and felt that an agility course or bike path would be a helpful addition to their playground to assist children with learning academic concepts, motor skill development, and to improve overall activity levels.

**Table 3:** Select Director and teacher survey responses for intervention satisfaction

Questions	Intervention ECE # 1	Intervention ECE # 2
***Stencil Use***		
Do providers use the playground to teach academic concepts through movement? If yes describe how?	Yes, they teach colors, numbers and letters using items on the playground.	Yes, especially now they have the stencils to be able to teach shapes, colors, numbers. Before they would play games outdoors that would teach counting, animals, colors, letters, and other concepts that sometimes went along with that week’s lesson.
Have you seen any change in students’ physical activity since the playground stencils were painted? If YES, describe how?	No.	Yes, they use them to bike or run or jump across regularly. Even if a teacher is not available to lead an activity, the children will make up their own games or will use the lines to run back and forth across the stencils or will hop, jump, skip, run from box to box.
Do teachers use the playground stencils to infuse physical activity into the school day outside of recess and lunch breaks?	No	No, but I like that idea and could see teachers doing this.
Which playground stencils have generated the most interest and/or activity?	All of them. The children play on them equally.	The teachers encourage them to all be used and the children love them. The bullseye may get used just a bit less because they have to have something to throw whereas the other two can be played on without a rock or bean bag, etc. If a teacher leads the activity, they have something to throw but if the kids play on it alone, we do not let them throw things on the playground.
***Future Directions***		
Do you have any recommendations for future training?	Provide the accessories needed for each activity (like bean bags). Maybe meet with teachers before school starts so it can be a group activity.	Our teachers liked that they all got their own book and could watch the video when they had the chance. Maybe a training where a child could be present to go through the activities to show the best way to instruct them on the different movements.
Do you have recommendations for future playground stenciling?	Paint stencils that do not need other equipment or provide the equipment.	We are interested in having more stencils painted now that we see how they can be used. We have tons of tricycles and push toys so having a bike path would be great. We would also like to do an agility course to help promote more motor skill development.
Additional Comments or Feedback	Staff was prepared each time they came and worked well with the teachers. The children were excited to have an addition to the playground. We are re-finishing our playground this Summer and may want to re-paint them once we are done. They will come in handy to use with our motor skills curriculum.	Wonderful job. The teachers and kids love the stencils.

## Discussion

This study investigated the effects of an ECE setting based environmental intervention on increasing PA, FMS, and reducing sedentary time in preschool-age children. PA behaviors did not increase after the implementation of the program in the intervention compared to the control groups. There was partial evidence that FMS performance significantly improved for the ECE centers who received the playground markings; however, this FMS improvement was not significant when compared to the control group. Children were actively engaged with the markings, and there were differences in which marking with which boys vs. girls more frequently engaged. ECE directors and teachers supported the implementation of these markings and the curriculum provided (both written and video) for the program. Overall, this investigation into a low-cost, outdoor playground enhancement was feasible and satisfactory, but could be improved for a significant effect on child PA and FMS.

To our knowledge, this is the first study that examined the effects of a playground stencil intervention among preschoolers’ PA and FMS performance. One study by Cardon and colleagues targeted PA improvements for preschoolers in a few ways, including playground markings, game equipment, and a combination of the two [30]. Results showed that these intervention strategies were not sufficient in changing PA behaviors during recess. However, that program did not investigate FMS, nor did they use guidance to enhance use of the playground markings. A systematic review echoed these findings and extended this discussion to older children, where mixed results in PA from playground marking interventions may be due to variability in availability in playground equipment (e.g., physical structures) or baseline PA behaviors [31]. For the present study, change in PA (e.g., total, moderate, vigorous, MVPA) or sedentary behaviors during the school day did not significantly differ for children in the intervention group versus control group [32].

There are several possibilities of why the lack of change in PA results were found. First, overall recess engagement was not captured during the six to eight weeks between measurements, so it is unclear if PA levels changed initially (perhaps due to a novelty effect) and then returned once this reactivity period subsided. Other research utilizing playground markings suggested that initial “novelty effects” might explain increases in PA for shorter interventions (i.e., < 4 weeks) [19, 30]. Second, intervention fidelity was not measured, particularly in reference to whether the curriculum integration occurred during recess. Directors and teachers reported that children primarily interacted with the stencils without teacher assistance; they also indicated portable playground equipment (e.g., bean bags, balls) or more teacher training may help in the future. Lastly, recruitment for centers that had available outdoor space for three stencils was challenging. Of the ~ 100 ECE centers that were screened, over half did not have adequate space to paint three stencils. It is unclear if ECE centers try to steer activity toward more built structures (e.g., climbing gyms, swing sets) or soft surfaces such as grass or rubberized coverings. Cardon and colleagues found that boys were more physically active on hard surfaces compared to girls on preschool playgrounds [33]. To date, there is no research on the amount of stencils that might elicit more PA, so a direction of future research is to examine if more stencils could have prompted more PA and more autonomy for children to engage in different types of PA.

In regards to FMS, the current study results indicate that change in ball skills, locomotor skills, and total FMS raw scores among preschoolers in the intervention group did not differ significantly with those in the control group. However, there was a significant improvement in ball skills and locomotor skills among preschoolers from the intervention group only. The mean of changes of ball skill and locomotor skill percentile scores after the intervention were slightly higher (but not at the level of statistical significance) among the preschoolers in the intervention group compared to those in the control group. Improvement of FMS is important to ensure children’s development trajectories of health [11, 34]. Evidence indicates that children with improved FMS are more physically active and fit [11, 35, 36]. Hence, FMS intervention in preschool setting is an effective strategy to improve preschoolers’ FMS [11, 37]. The small sample size of this study could affect the study results of not finding any significant difference between groups due to the limitation of being unable to remove variables from other factors that may be including the outcomes. Free play movement programs often improve children’s locomotor skills but not ball skills [38]. Therefore, it is important to facilitate combination of skill demonstrations and equipment or environmental prompts to engage preschoolers to improve locomotor and ball skills [11]. In addition, preschool teachers (non-motor experts) are often able to effectively teach children to improve ball skills in ECE [39]. In older children (10–16 years), revitalizing playgrounds had a limited impact on improving PA levels in children who were the least-active ahead of the renovations, indicating that upgraded environments alone seem to have limited impact when implemented without additional teacher or child support [40]. Placing stenciling indoors may also increase uptake and usage, as results from a study focused on indoor painted playground indicated that preschoolers in the intervention group spend more time performing specific activities such as standing, walking, running, and jumping/skipping [41].

One strength of this study is that painted markings on a playground is a pragmatic, low-cost intervention. As playground equipment is generally expensive and portable equipment may be damaged or lost over time, centers might find that enhancing playgrounds in this semi-permanent way may contribute to more opportunities for outdoor play at a lower cost. Directors and teachers indicated that students used the markings to reinforce academic concepts, which has more additive benefits for programs to reinforce both PA and academics in this population.

Limitations include the small sample for this pilot study. Only four schools were examined in one region of the United States, so results may only be generalizable to this area or to programs with similar characteristics. Children and teachers may use playground markings differently across varied outdoor environments such as urban vs. rural settings, in areas of high pollution, or in areas that experience extreme weather, such as heat or heavy snow. The main barrier to recruiting centers was the lack of adequate concrete space or concrete space that was limited (i.e., sidewalks, space against the buildings, or small bike paths, or concrete space that was not within safe confines like a fence or playground). The limited access to concrete space in most ECE centers is a limitation that must be considered for the expansion of this intervention. During the recruitment period, it was observed that rural ECE centers were more likely to have larger spaces, access to concrete spaces, and be under-resourced. Therefore, expansion may be better suited to rural areas and to smaller centers.

Finally, intervention fidelity was not measured, nor was PA measured immediately after introducing the markings on the playground. The goal was to ensure that enough time had elapsed to avoid any “novelty effect” (i.e., four weeks based on previous research) that might have accompanied this playground enhancement. Therefore, an investigation of the time course of children’s engagement with painted playgrounds would be helpful in planning future interventions. Extending the follow-up observations past the 6–8 weeks used in the present study might have allowed for reactivity to subside and allowed for additional FMS practice. Future work could extend the follow-up assessments to determine whether or not more time might have influenced either PA or FMS with additional recess practice time or if adding new markings as interest and trends change elicits continued PA. Additionally, a brief playground observation was used to see how children were engaging with the markings. Results showed that most of the play was child-led, the majority of activities took place in small groups, and boys and girls tended to engage with different markings. Teachers may be able to better integrate classroom lessons into the playground to increase use, as teacher engagement influenced children’s use of the stencils and engagement in PA. In addition, more insight from teachers and directors would be useful in understanding the dynamics surrounding this type of environmental change. More observations during the first few weeks may have provided additional insight into how to maximize these markings on the playground and how to better integrate PA opportunities during outdoor play time.

## Conclusions

Future studies should continue to examine the possibility of integrating low-cost interventions into the preschool environment with larger and more diverse samples in different regions and extending this work into advanced levels of behavioral intervention development (i.e., efficacy, effectiveness, implementation, and dissemination stages) [42]. There is also an opportunity to integrate the painted playgrounds with portable equipment, which has been previously shown to increase PA in some populations. Evaluating teacher engagement and intervention fidelity and curriculum items used would provide more insight into further modifications to this program. Finally, long-term changes, continued follow-up, as well as larger, well controlled trials will provide evidence on the effectiveness of these types of low-cost playground enhancements for both PA and FMS in preschool-age children.

## Data Availability

The datasets generated and/or analysed during the current study are not publicly available due to lack of informed consent for data sharing at the time of collection, but are available from the corresponding author on reasonable request.

## Abbreviations

PA: physical activity
MVPA: moderate-to-vigorous physical activity
FMS: fundamental motor skills
TGMD-3: Test of Gross Motor Development – 3rd edition
ECE: early childhood education
